# Psychosocial experiences of prostate cancer survivors after treatment: a systematic review of qualitative studies

**DOI:** 10.3389/fpubh.2025.1625611

**Published:** 2025-07-24

**Authors:** Junlian Xiang, Lifei Dai, Lin Tan, Dong Lv, Yongbo Chen, Liangyou Tang, Jiali Zhang, Xin Yi, Xiaoli Liu

**Affiliations:** ^1^Department of Urology, Deyang People’s Hospital, Deyang, China; ^2^School of Basic Medical Sciences and School of Nursing, Chengdu University, Chengdu, China; ^3^Department of Proctology, Deyang People’s Hospital, Deyang, China

**Keywords:** prostatic neoplasms, psychosocial factors, post-treatment, qualitative research, systematic review

## Abstract

**Background:**

Prostate cancer, the second most common male malignancy worldwide, treatment-related complications cause both physical dysfunction and psychosocial sequelae, significantly impairing quality of life. Now requires integrated biopsychosocial rehabilitation beyond disease-focused treatment, comprehensive assessment of psychosocial adaptation and illness perception is essential for developing evidence-based, patient-centered rehabilitation strategies to optimize post-therapy recovery.

**Objective:**

This study aims to systematically review and synthesize qualitative evidence on post-treatment psychosocial experiences in prostate cancer patients, thoroughly analyze patients’ lived experiences and coping strategies, and provide an evidence-based foundation for establishing a tiered psychosocial support system and developing clinical intervention protocols.

**Methods:**

This systematic review followed the Joanna Briggs Institute (JBI) methodology for qualitative meta-aggregation, with reporting structured according to the ENTREQ statement. Evidence was graded using the ConQual approach and critically appraised with the JBI Qualitative Assessment and Review Instrument (JBI-QARI). We systematically searched six major English databases for qualitative or mixed-methods studies investigating psychosocial experiences in post-treatment prostate cancer patients, with the literature search updated to February 28, 2024. Two reviewers independently performed study selection, followed by collaborative thematic synthesis to identify core themes.

**Results:**

A total of 22 studies from 12 countries were included, with 65 findings extracted and categorized into 4 synthesized findings consisting of 8 distinct categories: (1) Psychological and emotional responses (fear and anxiety responses, depression and emotional distress); (2) Healthcare information and systemic barriers (disease-related information needs, barriers in healthcare systems); (3) Social support and interpersonal adaptation (support system needs, social role and relationship adaptation); (4) Internal adaptation and external actions (internal psychological adjustment, external coping behaviors).

**Conclusion:**

Prostate cancer survivors face multifaceted psychosocial challenges during post-treatment recovery, with psychological and social responses impacting rehabilitation outcomes. Inadequate social support systems and gaps in healthcare information emerge as major barriers to recovery. To address these issues, healthcare providers should enhance communication effectiveness, while policymakers need to strengthen social support networks, government and corporate sectors should implement targeted policies, and family members should provide empathetic understanding and active encouragement, collectively fostering comprehensive patient support.

**Systematic review registration:**

https://www.crd.york.ac.uk/PROSPERO/recorddashboard, CRD42024537363.

## 1 Introduction

Prostate cancer (PCa) is the second most frequently diagnosed malignancy in men worldwide and the sixth leading cause of cancer-related deaths (1, 2). Epidemiological projections indicate a rising global burden, with an estimated 2.3 million new cases and 740,000 deaths annually by 2040 (3). Current treatments, such as surgery, hormone therapy, and radiation, have improved survival rates. However, these treatments often cause long-term side effects, including urinary problems, sexual difficulties, bone pain, and extreme tiredness (4).

Beyond physical morbidity, psychosocial sequelae profoundly impact patient well-being, such as anxiety about their illness, depression, and concerns about masculinity (5). A study revealed that the prevalence rates of PCa post-treatment depression and anxiety were 18.44 and 18.49%, respectively (6), patients with high-risk PCa exhibited elevated risks of both major depressive disorder and suicide mortality compared to lower-risk counterparts (7). Major depressive disorder is associated with increased healthcare expenditures, diminished quality-adjusted life years, and reduced overall survival duration among PCa survivors, these challenges greatly reduce their quality of life and ability to engage in daily activities (8).

Several qualitative meta-syntheses have been conducted on related topics, but they present notable limitations. One such study focused exclusively on the experiences of female partners of PCa survivors, rather than examining the survivors’ own perspectives (9), while another meta-synthesis specifically examined psychosocial experiences among African American PCa survivors (10). However, with recent advancements in qualitative systematic review methodology and the establishment of rigorous quality appraisal standards, new primary qualitative studies have emerged investigating post-treatment psychosocial adaptation in broader PCa populations. This methodological evolution necessitates an updated systematic review incorporating contemporary evidence to provide a more comprehensive understanding of survivors’ experiences.

This study employed the Joanna Briggs Institute (JBI) methodology (11) for systematic reviews to comprehensively synthesize existing evidence on post-treatment psychosocial experiences in PCa patients. Our approach incorporated a rigorous credibility assessment to evaluate findings and grade the synthesized results, aiming to provide an evidence-based foundation for establishing a tiered psychosocial support system.

## 2 Methods

This systematic qualitative review utilized the meta-aggregation methodology developed by the JBI. Following established guidelines, the study was conducted in accordance with the Enhancing Transparency in Reporting the Synthesis of Qualitative Research (ENTREQ) checklist (12) (in Supplementary Material 1). The study protocol was prospectively registered in PROSPERO (registration number: CRD42024537363).

### 2.1 Search strategy

We followed the three-step search method recommended by the Joanna Briggs Institute (JBI). First, we searched PubMed and CINAHL to find key words in titles, summaries, and subject terms. Next, we conducted a comprehensive computer search of six important databases: PubMed, CINAHL, Web of Science, Embase, PsycINFO, and Cochrane Library. The search strategy incorporated both Medical Subject Headings (MeSH) and free-text terms, including: “Prostatic Neoplasms” “Neoplasms, Prostate” “Prostate Cancer” “Psychological Factors” “Psychological Side Effects” “Qualitative Research” and related terms. We looked for all qualifying studies about PCa patients’ emotional and social experiences after treatment. Finally, we checked the reference lists of included studies to find more relevant papers. Two researchers (LFD and LT) separately reviewed all titles, summaries, and full papers. When they disagreed, they asked a third researcher (JLZ) for help. We searched all records from when each database started until February 28, 2024, the search strategy is given in Supplementary Material 2.

### 2.2 Inclusion and exclusion criteria

#### 2.2.1 Inclusion criteria

Participants: Patients diagnosed with PCa who had undergone treatment (age >18 years), including surgical therapy, chemotherapy and radiotherapy, etc.

Phenomenon of Interest: Psychosocial experiences of PCa patients following treatment.

Context: Psychosocial experiences of participants across all settings (home, hospital, and community).

Study Design: Qualitative studies, including phenomenology, ethnography, grounded theory, and action research. Mixed-methods studies were also included if qualitative data were reported separately.

#### 2.2.2 Exclusion criteria

Studies only about family members or healthcare workers; Studies not in English; Reviews, case reports or letters to the editor; Studies without full text or missing important data.

### 2.3 Literature screening

Two graduate students trained in qualitative research (LFD and LT) independently screened and extracted the literature. When disagreements occurred, a third researcher (JLZ) made the final decision. We used EndNote 20 to remove duplicate records. First, we screened titles and abstracts to exclude studies that did not meet our requirements. Then, we conducted a second screening by reading full texts to select studies for final inclusion.

### 2.4 Quality appraisal

To ensure the reliability of studies included in this meta-synthesis, we used the standard JBI Critical Appraisal Checklist for Qualitative Research (JBI-QARI). The JBI-QARI evaluates studies based on the following criteria: congruity between research methodology and stated philosophical perspective, research objectives, data collection methods, representation and analysis of data, interpretation of results, statement locating the researcher culturally or theoretically, influence between the researcher and participants, ethical considerations, adequacy of research findings, and reasonableness of conclusions. Each study was evaluated using the following classification: “Yes”: The study fully satisfies all methodological requirements of the item; “No”: The study unequivocally fails to meet the item’s essential criteria; “Unclear”: Incomplete reporting precludes definitive judgment; “Not applicable”: The criterion is irrelevant to the study’s design. “Yes” = 1 point and “No”/“Unclear” = 0 points; “Not applicable” items were excluded from the total score calculation, the total possible score is 10 points. Two graduate students trained in qualitative research methods (LFD and LT) independently evaluated the methodological quality of included studies. Any disagreements were resolved through discussion or by arbitration from a third reviewer (JLZ). Only studies achieving a total score ≥6 points were included in the final analysis.

### 2.5 Data extraction and synthesis

Two independent researchers (LFD and LT) extracted data based on the JBI qualitative data extraction table, covering author, country, research method, data collection method, number of interviewees, phenomenon of interest, and theme extraction, and original research results and examples. If the research subjects included other cancer patients, family members, or healthcare workers, only data related to PCa patients were extracted. A second reviewer checked the data extraction, and consensus was reached through discussion. By maintaining consistent extraction criteria, a large amount of evidence relevant to the research purpose was obtained.

A meta-aggregation approach was used to synthesize the findings. Based on an in-depth understanding of the philosophical underpinnings and methodology of each qualitative study, the included literature was repeatedly read, translated, analyzed, and interpreted. The research findings were coded and similar findings were merged into new categories through a manual integration method.

By analyzing the links between the categories, we summarized them into final synthesized findings, and each synthesized finding was graded according to the ConQual approach (13). The ConQual assessment comprises dependability and credibility. The dependability evaluation specifically examines: (1) Congruity between research methodology and research questions/objectives. (2) Consistency between methodology and data collection methods. (3) Alignment between methodology and data representation/analysis. (4) Explicit statement of researcher’s cultural/theoretical positioning. (5) Addressing of mutual researcher-participant influence. Findings with 4–5 “Yes” responses remain unchanged (no adjustment to the original level), those with 2–3 “Yes” responses move down 1 level, and findings with 0–1 “Yes” responses move down 2 levels. Credibility is assessed at three levels: (1) Unequivocal (findings supported by irrefutable evidence). (2) Equivocal (findings with questionable evidence linkage). (3) Unsupported (findings lacking data support).

## 3 Results

### 3.1 Search outcomes

**Figure 1:**
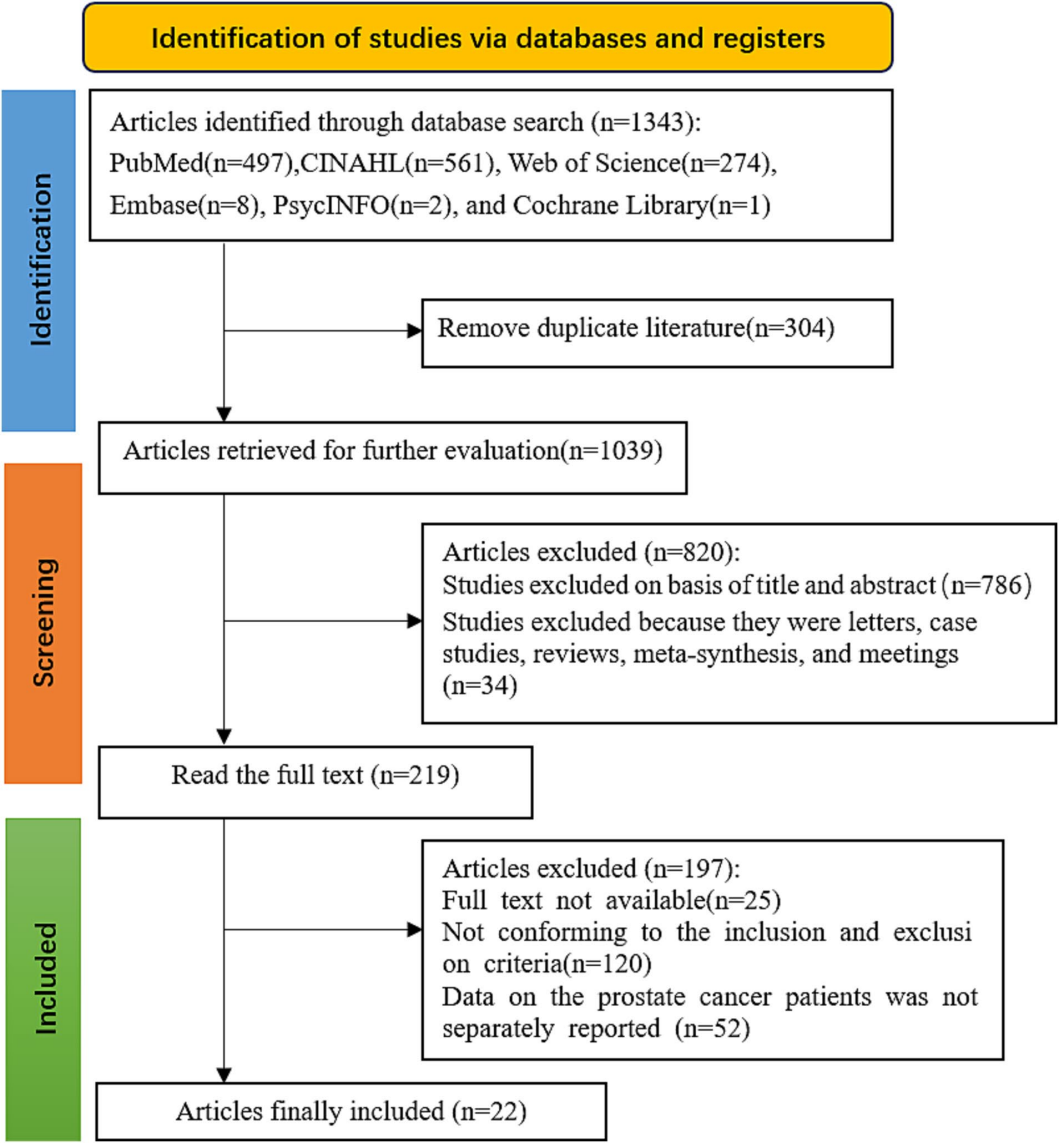
PRISMA flow diagram of the literature search and selection procedure for inclusion of qualitative studies.

A total of 1,343 studies were retrieved. After removing duplicates with EndNote 20, 1,039 remained. After screening titles and abstracts, 820 were excluded, leaving 219 for full-text screening. After reading the full texts, 197 were excluded. Finally, 22 studies were included, as shown in Figure 1.

### 3.2 Characteristics of included studies

The review included 22 studies (14–35) comprising 437 PCa patients across 12 countries: United Kingdom (*n* = 5), Japan (*n* = 4), Australia (*n* = 3), USA (*n* = 3), China (*n* = 3), Sweden (*n* = 2), Iran (*n* = 1), France (*n* = 1), Germany (*n* = 1), Canada (*n* = 1), Netherlands (*n* = 1), and Norway (*n* = 1). These comprised 1 grounded theory study and 21 phenomenological studies. Data collection methods were semi-structured interviews (*n* = 20) and focus group interviews (*n* = 2). The baseline characteristics of included studies are presented in Table 1. Study findings and their illustrations are detailed in Supplementary Material 3.

**Table 1:** Detailed characterization of the studies included in this systematic review.

Author	Year	Country	Category	Population	Phenomenon of interest	Themes extraction
Alexis et al. (23)	2023	United Kingdom	Phenomenological research	20	Supportive experiences of PCa patients after treatment	A1. Spiritual belief. A2. Questioning of male self-worth. A3. Desire for support from family and organizations
Adam et al. (25)	2023	United Kingdom	Phenomenological research	22	Treatment burden experience of PCa survivors	B1. Change in perception of treatment burden. B2. Fear of biopsy. B3. Take action to manage disease
Vyas et al. (22)	2022	United Kingdom	Phenomenological research	19	Experiences after radical treatment for PCa	C1. Reflecting on the illness. C2. Impacts interpersonal relationships. C3. Feeling distressed about sexual life
Matheson et al. (21)	2020	United Kingdom	Phenomenological research	28	Experience of psychological distress among PCa survivors	D1. Impacts masculinity, functioning, and connectedness. D2. Inability to control emotions
Bamidele et al. (24)	2019	United Kingdom	Constructivist grounded theory	25	Psychosocial experiences of PCa patients	E1. Gaining a sense of control over the disease. E2. Stigmatization. E3. Communication barriers between spouses. E4. Need to support family
Hayashi et al. (26)	2022	Japan	Phenomenological research	38	Experiences of sexual dysfunction following PCa treatment	F1. Decision conflict. F2. Loss of personal values. F3. Acceptance and Management of illness
Iguchi et al. (32)	2022	Japan	Phenomenological research	8	Fatigue and burden of PCa patients	G1. Avoiding social interaction. G2. Lack of doctor-patient communication
Akakura et al. (20)	2021	Japan	Phenomenological research	23	Experience of the impact of disease progression on daily physical activities	H1. Increased family burden. H2. Inadequate awareness of bone metastasis. H3. Dissatisfaction with treatment outcomes
Liu et al. (35)	2023	Australia	Phenomenological research	16	Work-related Experiences of PCa Survivors	I1. Efforts to resume normal work I2. Insufficient doctor-patient communication I3. Corporate support I4. Support from coworkers
Akakura et al. (19)	2020	Australia, Japan, China	Phenomenological research	12	Cognitive aspects of PCa patients in the Asia-Pacific region	J1. Concerns about expected lifespan J2. Insufficient understanding of the disease
Chambers et al. (31)	2018	Australia	Phenomenological research	28	The need for support to improve the quality of life for patients with advanced prostatic cancer	K1. Fear of the future, uncertainty. K2. Unwillingness to seek help. K3. Appeals being ignored. K4. Seeking solutions for side effects. K5. Shortage of medical resources in rural areas. K6. Economic burden of disease
Burbridge et al. (15)	2020	America, France, Germany	Phenomenological research	25	Emotional response experiences in patients with mCRPC	L1. Anxiety and fear. L2. Low Mood and depression. L3. Accept life-prolonging treatment
Holmstrom et al. (30)	2019	America	Phenomenological research	19	Symptoms and life impact in patients with mCRPC	M1. Changes in daily activities. M2. Keep a positive attitude. M3. Urinary frequency affects sleep quality
Tomaszewski et al. (17)	2017	America	Phenomenological research	19	Life experiences of PCa patients	N1. Feelings of depression and anxiety
Pan et al. (28)	2022	China	Phenomenological research	30	Experiences of treatment decision-making in PCa patients	O1: Limited decision participation. O2: Need disease knowledge and option autonomy
Wang et al. (33)	2022	China	Phenomenological research	13	Experiences of sexuality and intimacy after PCa treatment	P1. Adaptation of sexual behaviors and intimate relationships. P2. Inadequate sexual health support
Rönningå et al. (34)	2022	Sweden	Phenomenological research	11	Experience of PCa patients in the face of uncertainty regarding their disease status	Q1. Uncertainty about disease progression. Q2. Worrying about my family’s future
Doveson et al. (27)	2020	Sweden	Phenomenological research	16	Perspectives of PCa patients on life-extending treatments	R1. Consider life-prolonging treatment. R2. Reflection on life’s end. R3. Fear of uncontrolled symptoms at the end of life
Mardani et al. (14)	2023	Iran	Phenomenological research	12	Experience of PCa patients with disease recurrence	S1. Fear of not being fully cured. S2. Fear of cancer recurrence. S3. Efforts to adjust lifestyle to cope with the disease. S4. Relying on spiritual faith
Langelier et al. (16)	2022	Canada	Phenomenological research	15	PCa patients’ experiences of coping with masculinity	T1. Redefining masculinity. T2. Building a sense of control. T2. Maintaining social connections
van Ee et al. (18)	2018	Netherlands	Phenomenological research	22	Experience of older adult men diagnosed with PCa	U1. Afraid of chemotherapy. U2. Worried about PSA test results. U3. Reluctant to tell children. U4. Supported by grandchildren. U5.dissatisfied with nursing staff
Aunan et al. (29)	2021	Norway	Phenomenological research	16	PCa survivors’ experiences of the value of informational support	V1: Need for information and support. V2: Seeking peer support

### 3.3 Quality appraisal

The JBI-QARI quality assessment reports of the 22 studies showed that the scores for all 10 items ranged from 7 to 10. Most studies performed poorly on 2 items (Q6 and Q7). All 22 studies showed consistency between methodology and philosophical approach; 2 studies culturally or theoretically positioned the researcher. The quality assessment results are shown in Table 2.

**Table 2:** JBI QARI assessment results of included qualitative studies.

Author	Q1	Q2	Q3	Q4	Q5	Q6	Q7	Q8	Q9	Q10	Overall score
Alexis et al. (23)	Yes	Yes	Yes	Yes	Yes	Unclear	Yes	Yes	Yes	Yes	9
Adam et al. (25)	Yes	Yes	Yes	Yes	Yes	Unclear	Yes	Yes	Yes	Yes	9
Vyas et al. (22)	Yes	Yes	Yes	Yes	Yes	Unclear	Yes	Yes	Yes	Yes	9
Matheson et al. (21)	Yes	Yes	Yes	Yes	Yes	Unclear	Yes	Yes	Yes	Yes	9
Bamidele et al. (24)	Yes	Yes	Yes	Yes	Yes	Yes	Yes	Yes	Yes	Yes	10
Hayashi et al. (26)	Yes	Yes	Yes	Yes	Yes	Unclear	Yes	Yes	Yes	Yes	9
Iguchi et al. (32)	Yes	Yes	Yes	Yes	Yes	Unclear	Yes	Yes	Yes	Yes	9
Akakura et al. (20)	Yes	Yes	Yes	Yes	Yes	Unclear	Yes	Yes	Yes	Yes	9
Liu et al. (35)	Yes	Yes	Yes	Yes	Yes	Yes	Yes	Yes	Yes	Yes	10
Akakura et al. (19)	Yes	Yes	Yes	Yes	Yes	Unclear	Yes	Yes	Yes	Yes	9
Chambers et al. (31)	Yes	Yes	Yes	Yes	Yes	Unclear	Yes	Yes	Yes	Yes	9
Burbridge et al. (15)	Yes	Yes	Yes	Yes	Yes	Unclear	Unclear	Yes	Yes	Yes	8
Holmstrom et al. (30)	Yes	Yes	Yes	Yes	Yes	Unclear	Yes	Yes	Yes	Yes	9
Tomaszewski et al. (17)	Yes	Yes	Yes	Yes	Yes	Unclear	Unclear	Yes	Yes	Yes	8
Pan et al. (28)	Yes	Yes	Yes	Yes	Yes	Unclear	Yes	Yes	Yes	Yes	9
Wang et al. (33)	Yes	Yes	Yes	Yes	Yes	Unclear	Yes	Yes	Yes	Yes	9
Rönningå et al. (34)	Yes	Yes	Yes	Unclear	Yes	Unclear	Yes	Yes	Unclear	Yes	7
Doveson et al. (27)	Yes	Yes	Yes	Yes	Yes	Unclear	Yes	Yes	Yes	Yes	9
Mardani et al. (14)	Yes	Yes	Yes	Yes	Yes	Unclear	Unclear	Yes	Yes	Yes	8
Langelier et al. (16)	Yes	Yes	Yes	Yes	Yes	Unclear	Unclear	Unclear	Yes	Yes	7
van Ee et al. (18)	Yes	Yes	Yes	Yes	Yes	Unclear	Yes	Yes	Yes	Yes	9
Aunan et al. (29)	Yes	Yes	Yes	Yes	Yes	Unclear	Yes	Yes	Yes	Yes	9

JBI QARI appraisal instruments: Q1. Is there congruity between the stated philosophical perspective and the research methodology? Q2. Is there congruity between the research methodology and the research question or objectives? Q3. Is there congruity between the research methodology and the methods used to collect data? Q4. Is there congruity between the research methodology and the representation and analysis of data? Q5. Is there congruity between the research methodology and the interpretation of results? Q6. Is there a statement locating the researcher culturally or theoretically? Q7. Is the influence of the researcher on the research, and vice-versa, addressed? Q8. Are participants, and their voices, adequately represented? Q9. Is the research ethical according to current criteria or, for recent studies, and is there evidence of ethical approval by an appropriate body? Q10. Do the conclusions draw in the research report flow from the analysis, or interpretation, of the data?

### 3.4 Findings of the review

Systematic analysis of the 22 eligible studies (14–35) identified 65 outcomes, as presented in Table 3. Through inductive reasoning and integration of similar outcomes, 4 major integrated results were formed, comprising 8 categories: (1) Psychological and emotional responses (fear and anxiety responses, depression and emotional distress); (2) Healthcare information and systemic barriers (disease-related information needs, barriers in healthcare systems); (3) Social support and interpersonal adaptation (support system needs, social role and relationship adaptation); (4) Internal adaptation and external actions (internal psychological adjustment, external coping behaviors).

**Table 3:** Findings extracted from the included studies, categories and synthesized findings.

Findings	Categories	Synthesized findings
S1. Fear of not being fully cured S2. Fear of cancer recurrence L1. Anxiety and fear U1. Afraid of chemotherapy U2. Worried about PSA test results B2. Fear of biopsy R3. Fear of uncontrolled symptoms at the end of life K1. Fear of the future, uncertainty Q1. Uncertainty about disease progression Q2. Worrying about my family’s future	*Category 1: Fear and Anxiety Responses* Prostate cancer patients commonly experience intense fears of disease recurrence, treatment side effects, and future uncertainty.	*Synthesized finding 1: Psychological and Emotional Responses* Prostate cancer patients experience profound psychological distress characterized by fears of disease progression, treatment-related anxieties, and emotional disturbances.
L2. Low mood and depression N1. Feelings of depression and anxiety J1. Concerns about expected lifespan H3. Dissatisfaction with treatment outcomes D2. Inability to control emotions C3. Feeling distressed about sexual life	*Category 2: Depression and Emotional Distress* Prostate cancer patients develop depressive symptoms and emotional distress related to treatment outcomes, sexual dysfunction, and perceived loss of masculinity.	
J2. Insufficient understanding of the disease H2. Inadequate awareness of bone metastasis F1. Decision conflict O1. Limited decision participation O2. Need disease knowledge and option	*Category 3: Disease-Related Information Needs* Prostate cancer patients consistently demonstrate unmet needs regarding disease knowledge, treatment options, and prognostic information.	*Synthesized finding 2: Healthcare Information and Systemic Barriers* Inadequate health communication and systemic service barriers collectively create significant obstacles to optimal care acquisition for prostate cancer patients.
U5. Dissatisfied with nursing staff E2. Stigmatization K5. Shortage of medical resources in rural areas K6. Economic burden of disease G2. Lack of doctor-patient communication I2. Insufficient doctor-patient communication	*Category 4: Barriers in Healthcare Systems* The healthcare system presents structural barriers that compromise prostate cancer treatment efficacy.	
A3. Desire for support from family and organizations K3. Appeals being ignored P2. Inadequate sexual health support I3. Corporate support I4. Support from coworkers	*Category 5: Support System Needs* Patients with prostate cancer have multidimensional needs for family, social, and institutional support, yet they often face ignored support requests and insufficient sexual health support.	*Synthesized finding 3: Social Support and Interpersonal Adaptation* Prostate cancer patients face social network reconstruction, needing to sustain existing connections and adapt to disease-induced changes in intimacy and communication.
T3. Maintaining social connections U3. Reluctant to tell children U4. Supported by grandchildren H1. Increased family burden D1. Impacts masculinity, functioning, and connectedness C2. Impacts interpersonal relationships A2. Questioning of male self-worth E3. Communication barriers between spouses E4. Need to support family F2. Loss of personal values M1. Changes in daily activities M3. Urinary frequency affects sleep quality K2. Unwillingness to seek help G1. Avoiding social interaction P1. Adaptation of sexual behaviors and intimate relationships	*Category 6: Social Role and Relationship Adaptation* The disease impacts the gender identity, family roles, and personal value systems of prostate cancer patients.	
S4. Relying on spiritual faith T2. Building a sense of control A1. Spiritual belief C1. Reflecting on the illness E1. Gaining a sense of control over the disease B1. Change in perception of treatment burden R2. Reflection on life’s end M2. Keep a positive attitude	*Category 7: Internal Psychological Adjustment* Prostate cancer patients utilize spiritual faith for psychological adaptation, reflecting on their illness to regain a sense of control.	*Synthesized finding 4: Internal Adaptation and External Actions* Prostate cancer patients address disease-related challenges through a combination of internal psychological and spiritual adaptation, and external modifications in lifestyle and active engagement in support-seeking behaviors.
S3. Efforts to adjust lifestyle to cope with the disease L3. Accept life-prolonging treatment T1. Redefining masculinity B3. Take action to manage disease F3. Acceptance and management of illness R1. Consider life-prolonging treatment V2. Seeking peer support K4. Seeking solutions for side effects I1. Efforts to resume normal work	*Category 8: External Coping Behaviors* Prostate cancer patients utilize multifaceted external coping strategies for disease management.	

#### 3.4.1 Synthesized finding 1: psychological and emotional responses

##### 3.4.1.1 Category 1: fear and anxiety responses

As PCa progresses, patients exhibit progressive psychological deterioration characterized by an anxiety-fear complex. This includes fear of treatment outcomes, PSA monitoring-related anticipatory anxiety, and chemotherapy phobia (13). Invasive procedures such as transrectal prostate biopsy often induce significant pain, leading to decreased patient compliance in some cases.

“It was traumatic enough to say that I’m just sick of this idea of giving biopsies, seeing doctors. I even missed giving a PSA on the three monthly after May” (20).

With disease progression, patients face increasing risks of treatment failure and heightened uncertainty regarding survival outcomes, which may trigger profound anxiety about disease prognosis.

“Well, that’s only 3 months away, it’s only 2 months away actually. What’s going to happen between now and then” (26).

##### 3.4.1.2 Category 2: depression and emotional distress

Androgen deprivation therapy (ADT) induces endocrine alterations in PCa patients, causing feminizing changes, body image distress, and sexual dysfunction that leads to marked sexual dissatisfaction in many patients.

“I’m not happy about that at all because although I’m 76 you know, as I say, the desire is still very much there” (22).

Patients following radical prostatectomy commonly develop postoperative complications including urinary incontinence and sexual dysfunction. These sequelae often induce feelings of social discrimination and internalized stigma, which may progress to adjustment disorders characterized by diminished treatment satisfaction, emotional instability, and depressive symptoms.

“It’s just spontaneous. I just all of a sudden feel sad and want to cry” (21).

#### 3.4.2 Synthesized finding 2: healthcare information and systemic barriers

##### 3.4.2.1 Category 3: disease-related information needs

Patients demonstrated a strong desire for disease-related knowledge, most displayed limited awareness of PCa treatment options and their potential adverse effects, along with deficient understanding of disease symptomatology, clinical staging, and advanced-stage manifestations.

“The urologist said I could call if I had any questions after the consultation. I called, several times, but never got in touch. I was very disappointed…doctor is so busy and unavailable” (29).

Patients expressed clear preferences for shared decision-making with healthcare providers, emphasizing the need for comprehensive disease education and preservation of autonomous treatment choice, rather than physician-dominated decision-making.

“I was worried concerning future recurrence, I gave up on sexual function and chose total resection instead of nerve-sparing prostatectomy” (26).

##### 3.4.2.2 Category 4: barriers in healthcare systems

Deficiencies in healthcare systems adversely impact patient care experiences through multiple pathways, including impaired physician-patient communication and insufficient psychosocial support provision, ultimately diminishing patient satisfaction with both medical and nursing care.

“…and I thought that a nurse practitioner was someone who, well, who sympathized with you a bit, provided care, checked whether quicker treatments were possible. Well, the name says it all, practitioner” (18).

Patients with advanced PCa face major challenges during treatment, especially in rural areas with limited healthcare services. Many must travel long distances to receive care. Frequent hospital visits and treatments take up a lot of time and cause financial problems, creating extra stress for patients and their families (36).

“I’m up in the country and there was really no services in my town, an hour and a half trip to the closest place where could get anything done…They did not even have chemotherapy services here.” (31).

#### 3.4.3 Synthesized finding 3: social support and interpersonal adaptation

##### 3.4.3.1 Category 5: support system needs

Post-treatment PCa patients frequently struggle to resume normal social functioning, Urinary incontinence and muscle weakness exacerbate occupational challenges and interpersonal relationship maintenance. Despite expressing needs for multifaceted support, internalized shame and traditional masculinity norms deter patients from seeking help for sensitive issues like sexual dysfunction. Healthcare providers should proactively inquire about these unmet needs during follow-up consultations.

“… the doctors and nurses do not give the right support to the black community. Because when I started to seek advice and support, I could not find none, I could not find nothing” (23).

While financial pressures drive many PCa patients to urgently seek early return-to-work, occupation-dependent disparities exist: corporate employees benefit from organizational support enabling treatment compliance, whereas self-employed individuals face operational disruption and income loss due to lacking safeguards, highlighting the need for equitable workplace reintegration policies.

“We were able to work around the times when I had my immune system down, I was able to get support from other people within the group to carry out the work that I was going to do” (35).

##### 3.4.3.2 Category 6: social role and relationship adaptation

The multifaceted post-therapeutic symptomatology exerts profound impacts on patients’ psychosocial functioning and interpersonal dynamics. Sexual dysfunction frequently precipitates dyadic intimacy impairment and conjugal communication deficits.

“…the relationship wasn’t that great…the sexual aspect of things went out of the window… I feel that there’s something missing, and sort of when am I going to get that back…” (24).

Concurrently, urinary incontinence and treatment-related physical changes may trigger masculine identity conflict, leading to negative self-perception, reduced healthcare engagement, and social withdrawal behaviors.

“Going out has become troublesome… I have stopped going to those completely. I do not even want to go outside anymore” (32).

Some patients demonstrated improved psychosocial adaptation through family support systems, particularly spousal and intergenerational relationships (18). These support networks facilitated adaptive coping strategies that enhanced intimate partner connections and thereby reduced symptom-related psychosocial distress.

“We do not need to talk about this, as she knows my situation [erectile dysfunction]…. Our relationship is good so that we do not need any communication, she understands this.” (33).

#### 3.4.4 Synthesized finding 4: internal adaptation and external actions

##### 3.4.4.1 Category 7: internal psychological adjustment

Patients adapt to disease challenges through internal psychological mechanisms, including therapeutic cognition reappraisal (25), religious or spiritual comfort-seeking.

“I believe that, I have to do my own part and God will do his own part because God heals it. He’s the one that can heal me” (23).

Through the process of illness reflection, patients reconstruct their understanding of life’s finitude to reconcile with disease prognosis, thereby maintaining a sense of control over their condition.

“…cost what it will cost, I have to take charge of my life, I have to be looked up to again as a man…” (24).

##### 3.4.4.2 Category 8: external coping behaviors

In disease management, PCa patients employ not only internal psychological adjustments but also external behavioral adaptation strategies, including active participation in life-prolonging therapies and deliberate lifestyle modifications (14).

“In no way have I imagined that I will all of a sudden be completely cured. I can … keep going and feel well a little longer than I would otherwise have done” (27).

Peer support allows PCa patients to share lived experiences, work together to manage complications arising from treatment, and psychologically reconceptualize masculine identity.

“…talking to others in the same situation, how others have experienced getting PCa and side effects…I think it’s very reassuring to talk to others who have been through the same” (29).

### 3.5 Evidence quality evaluation results

This study evaluated the quality of four synthesized findings using the ConQual approach, with all results maintaining a moderate evidence grade (B), as shown in Table 4. For dependability, all met the 4–5 “Yes” criteria and thus required no downgrade. For credibility, all findings except “Internal Adaptation and External Actions” (remained unchanged) were downgraded by one level. Ultimately, all four synthesized findings were rated as moderate (B), indicating they possess moderate levels of credibility and dependability.

**Table 4:** ConQual summary of findings.

Synthesized finding	Type of research	Dependability	Credibility	ConQual score
Psychological and Emotional Responses	Qualitative	No change (scored 5/5 for the 5 criteria in 1 study, 4/5 in 8 studies,3/5 in 3 studies)	Downgrade one level*	Moderate (B)
Healthcare Information and Systemic Barriers	Qualitative	No change (scored 5/5 for the 5 criteria in 2 studies, 4/5 in 8 studies)	Downgrade one level*	Moderate (B)
Social Support and Interpersonal Adaptation	Qualitative	No change (scored 5/5 for the 5 criteria in 2 studies, 4/5 in 10 studies,3/5 in 1 studies)	Downgrade one level*	Moderate (B)
Internal Adaptation and External Actions	Qualitative	No change (scored 5/5 for the 5 criteria in 2 studies, 4/5 in 8 studies,3/5 in 3 studies)	Remains unchanged	Moderate (B)

*The evidence level was downgraded due to methodological heterogeneity or quality variations.

## 4 Discussion

### 4.1 Correctly viewing the disease progression, regulating negative emotions

The findings of this study demonstrate that PCa patients commonly experience multiple psychosocial distresses, including disease-related anxieties, PSA test-related anxiety, future uncertainty, depressive symptoms, emotional dysregulation, treatment dissatisfaction, life expectancy concerns, and sexual dysfunction.

Healthcare professionals are critical in helping them maintain a correct mindset toward the progression of the illness and actively manage negative emotions. Anxiety about abnormal PSA levels and fear of cancer recurrence are significant psychological challenges for PCa survivors after treatment (37, 38). Healthcare professionals should educate patients to correctly perceive PSA levels and shift their focus from merely reducing levels to enhancing their overall well-being. The decision-making process should focus on communication to build patient trust in the medical staff and mitigate uncertainty-associated fear.

Incorporating spiritual beliefs can increase patients’ morale and regulate their negative emotions. A study in Iran highlighted that some patients use spiritual beliefs to cope with fears of an incomplete cure and cancer recurrence (14), self-efficacy helps improve depression and anxiety in patients undergoing radical prostatectomy (39). Sharing life experiences with peers, engaging in social activities, and engaging in healthy behaviors are critical (40), whether through face-to-face interactions, phone calls, online chats, or meetings, provides patients with real insights and enables them to maintain optimistic attitudes and overcome stigmatization (41). Psychological care interventions can positively affect patients’ depression and anxiety (42), healthcare providers should integrate spiritual care into their practices to enhance emotional well-being.

Empowering patients is vital for understanding the disease, boosting their psychological health, and reducing uncertainty regarding disease progression. Decision aids can effectively reduce conflict, enhance understanding of the disease and treatment options, and increase risk awareness and satisfaction (43). Canadian researchers conducted a six-month PCa empowerment program (PC-PEP) (44), focusing on providing health education, empowerment, dietary advice, and social support, which notably reduced the psychological impact of treatment-related side effects of PC-PEP. Another PC-PEP initiative adopted a multifaceted approach through online and real-time interactions to empower the patients (45). Empowerment addresses biopsychosocial needs at various treatment stages, and enhances psychological well-being.

### 4.2 Optimizing symptom management plans to alleviate symptom burden

The findings of this qualitative meta-synthesis indicate that PCa patients develop lifestyle modifications, social disengagement, compromised interpersonal relationships secondary to disease manifestations, and treatment-induced complications. In response, patients adopt comprehensive adaptive approaches encompassing behavioral adjustments, gender role redefinition, illness self-regulation, peer support, and cognitive-emotional adaptation to mitigate these impacts.

Post-treatment PCa patients require prompt restoration of daily functioning, necessitating systematic symptom management interventions (46). Healthcare professionals should implement enhanced symptom management protocols and improve clinician understanding of patient experiences through personalized discharge plans, lifestyle guidance, and self-management education to reduce symptom burden. Structured exercise programs provide multisystem benefits, counteracting androgen deprivation therapy complications (47), improving incontinence recovery post-prostatectomy (48), potentially modulating PSA dynamics (49), and enhancing overall physical, psychological, and social well-being (50–52). These integrated approaches collectively optimize quality of life and functional recovery.

Post-prostatectomy urinary incontinence significantly affects health-related quality of life (HRQOL). Conventional conservative treatments include pelvic floor muscle training, biofeedback, and electrical stimulation (48). Proper guidance and supervision of pelvic floor exercises can shorten the recovery time (53), a systematic review demonstrated that preoperative pelvic floor muscle training can improve urinary incontinence at 3 months following radical prostatectomy (54). Patients with indwelling catheters require education on catheter maintenance for drainage patency and catheter-associated urinary tract infections prevention, alongside nocturnal fluid restriction to reduce nocturia.

Fatigue is another critical issue that can hinder daily and social activities, and some patients report that it pushes them toward depression (32). Patients affected by fatigue should reduce their activity levels, switch to less demanding jobs, or take breaks before returning to work to aid physical recovery (35). Those who need care from spouses or children may feel uncomfortable changing their family role, it is important to accept help from family members and life-prolonging treatments, and plan together to complete previously enjoyable activities.

Patients experiencing sexual dysfunction (SD) feel lonely, and their partners are unwilling to understand their SD issues or unable to tolerate sexless relationships (55). They should correctly understand body changes without feeling guilty about disruptions in their sexual lives. Researchers have explored alternative methods for achieving sexual pleasure without clinical intervention, such as oral stimulation by a partner, which can be satisfactory, even without full erection (24). The healthcare system provides psychosexual support and offers guidance on how to navigate marital communication (24). Such support can improve the quality of communication between spouses, thereby enhancing the overall QoL. Music therapy or prescribed pain medications can be effective in pain management. Music therapy has shown benefits in reducing anxiety, depression, and pain in cancer patients (56, 57), its application during prostate biopsies has been shown to alleviate both pain and procedural stress.

### 4.3 Improving the social support system to safeguard patient interests

This systematic review reveals PCa patients face multifaceted challenges including disease knowledge deficits, decision-making conflicts with limited autonomy, and communication barriers exacerbated in resource-limited settings. Additional burdens encompass financial toxicity, social stigmatization, and dissatisfaction with care quality. These findings substantiate the imperative for implementing shared decision-making protocols and establishing psychosocial support systems.

Healthcare providers should provide comprehensive explanations of treatment options, including the benefits, limitations, and mechanisms of both surgery and androgen deprivation therapy (ADT), as misunderstandings may compromise therapeutic adherence (58). One patient mentioned that post-treatment support from healthcare professionals can significantly boost confidence (35), and improving the perioperative management model for PCa can help enhance patients’ mental states, quality of life, and self-care abilities (59). Nurses who provide emotional support, information, planning and follow-up, and comprehensive assessments of patients’ conditions are essential to PCa care team (60). A clinical study demonstrated that compassion-centered spiritual care significantly reduced depression levels (61), while systematic evidence confirms the need to integrate spiritual care into the management of patients with serious illnesses (62). Healthcare workers should empathize with patients with PCa to understand their emotions and high-quality service attitudes. For patients in rural or remote areas or those who cannot frequently visit medical facilities because of poor health, health care providers can employ online or telephone services to monitor post-discharge health.

Population aging and healthcare workforce shortages necessitate medical robotics innovation, robotic-assisted systems enhance psychological intervention efficacy and service quality. German hospitals use robots to assist early postoperative patient activities (63). In Switzerland, the Lio mobile robot performs autonomous disinfection and assists staff and patients (64). Robots can be used in psychological health interventions for patients with anxiety or depression. A meta-analysis in the UK found that social assistive robots might improve patients’ loneliness, stress, and pain and alleviate pain throughout their life cycle (65). Robots, such as Paro, have been used to improve psychological symptoms, reduce medication usage, and enhance social interactions among the older adult (66). Such robots could benefit patients with PCa by providing psychological counseling and assessing the therapeutic effects.

Governments and businesses must support patients’ employment needs by understanding their physical limitations, providing suitable job positions, and establishing cancer support centers and community groups (35). Regular organizations for early PCa screening and popularization of knowledge on cancer prevention are urgently needed. Governments should support “living with cancer” initiatives, such as establishing agencies such as the Fair Employment Opportunity Commission, tax relief for businesses employing cancer patients (35) and developing policies to encourage healthcare professionals from large hospitals to offer free clinics in remote areas, ensuring that patients in underserved locations receive consistent and high-quality healthcare.

### 4.4 Limitations

Considering the limitations of this study, these results should be interpreted with caution: (1) Only 22 qualitative studies were included in this analysis; (2) All studies were conducted in just 12 countries and restricted to English-language publications; (3) The majority of the included qualitative studies demonstrated moderate methodological rigor (scoring 7–9 points). These limitations hinder a comprehensive understanding of the psychosocial experiences of PCa patients. We hope future research will incorporate more qualitative studies to explore the psychosocial dimensions of PCa patients’ experiences.

## 5 Conclusion

This systematic review of qualitative studies examines the psychosocial experiences of PCa survivors across different countries. The findings indicate that treatment complications, healthcare system barriers, and insufficient social support often lead to psychological distress and reduced quality of life. Healthcare professionals should address both physical and psychological needs while providing emotional support and coping strategies. The adoption of innovative care technologies is recommended to enhance efficiency and improve service delivery. Policy support from governments and businesses remains crucial to safeguard patients’ livelihood, security, and employment needs. Family members play an important role by fostering positive emotions through improved communication and facilitating reintegration into daily life.
