# Supplementary material for: Psychosocial experiences of prostate cancer survivors after treatment: a systematic review of qualitative studies
Source: Front Public Health. 2025 Jul 24;13:1625611. doi: 10.3389/fpubh.2025.1625611 (PMC12328169; doi:10.3389/fpubh.2025.1625611)
Supplement: Supplementary file 2 [file Supplementary_file_2.docx]

**Database Search Strategy**

**PubMed:**

( ( "Prostatic Neoplasms"[Mesh] OR "Neoplasms, Prostatic"[Title/Abstract] OR "Neoplasm, Prostatic"[Title/Abstract] OR "Prostatic Neoplasm"[Title/Abstract] OR "Prostate Neoplasms"[Title/Abstract] OR "Neoplasms, Prostate"[Title/Abstract] OR "Neoplasm, Prostate"[Title/Abstract] OR "Prostate Neoplasm"[Title/Abstract] OR "Prostate Cancer"[Title/Abstract] OR "Cancer, Prostate"[Title/Abstract] OR "Cancers, Prostate"[Title/Abstract] OR "Prostate Cancers"[Title/Abstract] OR "Cancer of Prostate"[Title/Abstract] OR "Cancer of the Prostate"[Title/Abstract] OR "Prostatic Cancer"[Title/Abstract] OR "Cancer, Prostatic"[Title/Abstract] OR "Cancers, Prostatic"[Title/Abstract] OR "Prostatic Cancers"[Title/Abstract] ) AND ( "Psychology"[Mesh] OR "Psychological Factors"[Title/Abstract] OR "Factor, Psychological"[Title/Abstract] OR "Psychological Factor"[Title/Abstract] OR "Factors, Psychological"[Title/Abstract] OR "Side Effects, Psychological"[Title/Abstract] OR "Psychological Side Effect"[Title/Abstract] OR "Psychological Side Effects"[Title/Abstract] OR "Psychosocial Factors"[Title/Abstract] OR "Factor, Psychosocial"[Title/Abstract] OR "Factors, Psychosocial"[Title/Abstract] OR "Psychosocial Factor"[Title/Abstract] OR "Psychosocial Support Systems"[Mesh] OR "Adaptation, Psychological"[Mesh] OR "Quality of Life"[Mesh] OR "Emotions"[Mesh] OR "Anxiety"[Mesh] OR "Depression"[Mesh] OR "Fear"[Mesh] OR "Stress, Psychological"[Mesh] OR "Self Concept"[Mesh] OR "Social Support"[Mesh] OR "Interpersonal Relations"[Mesh] OR "Family Relations"[Mesh] OR "Masculinity"[Mesh] OR "Social Stigma"[Mesh] ) AND ( "Qualitative Research"[Mesh] OR "Research, Qualitative"[Title/Abstract] OR "Narrative Medicine"[Mesh] OR "Interviews as Topic"[Mesh] OR "Focus Groups"[Mesh] OR "Grounded Theory"[Mesh] OR "Thematic Analysis"[Title/Abstract] OR "Content Analysis"[Title/Abstract] OR "Qualitative Study"[Title/Abstract] OR "Qualitative Studies"[Title/Abstract] OR "Qualitative Method*"[Title/Abstract] OR "Qualitative Interview*"[Title/Abstract] OR "Qualitative Data"[Title/Abstract] OR "Qualitative Analysis"[Title/Abstract] ) )

**CINAHL:**

((MH "Prostatic Neoplasms+" OR TI "Prostatic Neoplasms" OR AB "Prostatic Neoplasms" OR TI "Neoplasms, Prostatic" OR AB "Neoplasms, Prostatic" OR TI "Prostatic Neoplasm" OR AB "Prostatic Neoplasm" OR TI "Prostate Neoplasms" OR AB "Prostate Neoplasms" OR TI "Prostate Cancer" OR AB "Prostate Cancer" OR TI "Cancer of Prostate" OR AB "Cancer of Prostate" OR TI "Cancer of the Prostate" OR AB "Cancer of the Prostate" OR TI "Prostatic Cancer" OR AB "Prostatic Cancer") AND ((MH "Psychology+" OR MH "Psychological Factors+" OR MH "Adaptation, Psychological+" OR MH "Quality of Life+" OR MH "Emotions+" OR MH "Anxiety+" OR MH "Depression+" OR MH "Fear+" OR MH "Stress, Psychological+" OR MH "Self Concept+" OR MH "Social Support+" OR MH "Interpersonal Relations+" OR MH "Family Relations+" OR MH "Masculinity+" OR MH "Social Stigma+") OR (TI "Psychological Factors" OR AB "Psychological Factors" OR TI "Psychosocial Factors" OR AB "Psychosocial Factors" OR TI "Psychosocial Factor" OR AB "Psychosocial Factor")) AND ((MH "Qualitative Studies+" OR MH "Grounded Theory+" OR MH "Phenomenological Research+" OR MH "Focus Groups+" OR MH "Interviews+" OR MH "Content Analysis+" OR MH "Thematic Analysis+") OR (TI "Qualitative Research" OR AB "Qualitative Research" OR TI "Qualitative Study" OR AB "Qualitative Study" OR TI "Qualitative Studies" OR AB "Qualitative Studies" OR TI "Qualitative Method*" OR AB "Qualitative Method*" OR TI "Qualitative Interview*" OR AB "Qualitative Interview*" OR TI "Thematic Analysis" OR AB "Thematic Analysis" OR TI "Content Analysis" OR AB "Content Analysis")))

**Web of Science:**

(TI=("prostatic neoplasms" OR "prostate cancer" OR "prostatic cancer") OR AB=("prostatic neoplasms" OR "prostate cancer" OR "prostatic cancer") OR AK=("prostatic neoplasms" OR "prostate cancer" OR "prostatic cancer") OR KP=("prostatic neoplasms" OR "prostate cancer" OR "prostatic cancer"))AND(TI=("psychology" OR "psychosocial" OR "quality of life") OR AB=("psychology" OR "psychosocial" OR "quality of life") OR AK=("psychology" OR "psychosocial" OR "quality of life") OR KP=("psychology" OR "psychosocial" OR "quality of life"))AND(TI=("qualitative research" OR "qualitative study" OR "qualitative method*") OR AB=("qualitative research" OR "qualitative study" OR "qualitative method*") OR AK=("qualitative research" OR "qualitative study" OR "qualitative method*") OR KP=("qualitative research" OR "qualitative study" OR "qualitative method*"))

**Embase:**

(TI "prostate cancer" OR TI "prostatic neoplasms" OR TI "neoplasms, prostatic" OR TI "prostatic neoplasm" OR TI "prostate neoplasms" OR TI "neoplasms, prostate" OR TI "prostate neoplasm" OR TI "cancer, prostate" OR TI "prostate cancers" OR TI "cancer of prostate" OR TI "cancer of the prostate" OR TI "prostatic cancer") AND (TI "psychology" OR TI "psychological factor" OR TI "psychosocial factor" OR TI "psychosocial support" OR TI "psychological adaptation" OR TI "quality of life" OR TI "emotion" OR TI "anxiety" OR TI "depression" OR TI "fear" OR TI "mental stress" OR TI "self concept" OR TI "social support" OR TI "interpersonal communication" OR TI "family relation" OR TI "masculinity" OR TI "social stigma" OR TI "psychological factors" OR TI "psychosocial factors") AND (TI "qualitative research" OR TI "meta synthesis" OR TI "narrative medicine" OR TI "interview" OR TI "focus group" OR TI "grounded theory" OR TI "thematic analysis" OR TI "content analysis" OR TI "qualitative study" OR TI "qualitative studies" OR TI "qualitative method*" OR TI "qualitative interview*" OR TI "qualitative data" OR TI "qualitative analysis")
