# Supplementary material for: Psychosocial experiences of prostate cancer survivors after treatment: a systematic review of qualitative studies
Source: Front Public Health. 2025 Jul 24;13:1625611. doi: 10.3389/fpubh.2025.1625611 (PMC12328169; doi:10.3389/fpubh.2025.1625611)
Supplement: Supplementary file 3 [file Supplementary_file_3.docx]

Table1 Findings extracted from the included studies and their illustrations

| **Author，year** | **Numbered findings** | **Illustrations** |
| --- | --- | --- |
| Obrey, et al (2023) | A1.Spiritual belief  A2.Questioning of male self-worth  A3.Desire for support from family and organizations | A1.“I believe that, I believe it. That’s why I said, I have to do my own part and God will do his own part because God heals it. He’s the one that can heal me. You can do anything, you can, the doctor will do their part, you will do your part, but it is God that sanctions it, that’s my belief.”(P4)  A2.“I knew I wouldn’t have semen…To have something then taken away from you, that is like life itself. And then to not have it, that itself is something that mucks with your head anyway, to be honest with you, And I probably, I’m about a six and a half, seven on depression, out of ten, I’m about there.”(P3)  A3.“People give up because they don’t understand what’s going on. And the doctors and nurses do not give the right support to the black community. Because when I started to seek advice and support, I couldn’t find none, I couldn’t find nothing.” (P4) |
| Rosalind, et al(2023) | B1.Change in perception of treatment burden  B2.Fear of biopsy  B3.Take action to manage disease | B1.“We stay out in the countryside so every trip into the hospital takes so much longer, it’s like if you’re going to the hospital for an appointment that’s really your day taken up. (…) I don’t regard it as an inconvenience, I don’t resent it, it doesn’t get me annoyed or anything like that, I just think, ‘Oh, it’s something I’ve got to do’. Yeah, time passes so quick when you’re older, it won’t be long before you’re back home anyway.”(P7)  B2.“I haven’t seen a doctor, I don’t want to see a doctor. (…) it was traumatic enough to say that I’m just sick of this idea of giving biopsies, seeing doctors. And I even chickened out, I don’t know if that’s the word, I even missed giving a PSA on the three monthly after May.”(P8)  B3.“Cancer management. Certainly, if you want to put it in terms of a graph, the graph has gone up steadily and quite steeply immediately post operation, but it has levelled out and is now on the way down and I’m pleased with that.”(P5) |
| Neel, et al (2022) | C1. Reflecting on the illness  C2. Impacts interpersonal relationships  C3. Feeling distressed about sexual life | C1.“Yes, that’s exactly what it is. That is, it is coming back. And I suppose, again, in hindsight, I think I benefit from having the operation of having the prostate removed completely.”(P8)  C2.“Think I felt pressure from my partner as well, because I didn’t feel they were being particularly supportive. They were trying to be in their way. But it wasn’t really being supportive because it was making me question everything that the health profession was trying to do for me.”(P9)  C3.“Well, obviously, I’m not happy about that at all because although I’m seventy-six you know, as I say, the desire is still very much there, but it’s a price you have to pay.”(P11) |
| Lauren, et al(2020) | D1.Impacts masculinity, functioning, and connectedness  D2.Inability to control emotions | D1(1). “It’s just not right. It’s the self-esteem, not feeling like a complete man . . . not just the sex side of things, it’s the strength to do things. You’re trying to lift things that you used to think nothing of before, and you can’t do it anymore.”(P322)  D1(2).“The confidence hasn’t come back. So life’s quieter these days. I used to do a lot of [theater work] . . . and I’ve stopped doing that completely, because I don’t have the confidence to do it anymore. So the social side of my life has diminished.” (P323)  D2.“It’s just spontaneous. I just all of a sudden feel sad and want to cry.”(P323) |
| Olufikayo, et al(2019) | E1.Gaining a sense of control over the disease  E2.Stigmatization  E3.Communication barriers between spouses  E4.Need to support family | E1.“...but I need to earn my money, I mean you are already taking my manliness away, taking everything away from me, you are stripping me bit by bit, right? I just thought no... cost what it will cost, I have to take charge of my life, I have to be looked up to again as a man...”(P1716)  E2.“But er, you know with we Africans when they say you are not a man, it means er, one thing that comes to mind is you can't have children, you see when you're not a man, you can't have children...in my own case even though I could still have some activity, you know when they remove the prostate, that's it, you know, you don't have any more semen, you see... I feel bad about it, I feel bad about it, but at least I'm alive you know...”(P1716)  E3.“...I felt the burden because I am somebody that talks... you know that he was always quiet... I don't know what is on your mind...you know the relationship wasn't that great...the sexual aspect of things went out of the window...so yeah that's the main issue now because it's not easy...I ask myself is it going to be like this forever? Or is it going to correct itself...emotionally I feel that there's something missing, and sort of when am I going to get that back...you feel a bit frustrated sometimes...” (P1716)  E4“...because I need to be there for my family, I need to be able to provide for my family...I have to, as a man, look to my family, ok, I have to, if I am not able to have that ability to provide by myself, then mentally, it is destroying me, it will destroy me...” (P1716) |
| Saeko, et al (2022) | F1.Decision conflict  F2.Loss of personal values  F3.Acceptance and management of illness | F1.“I was worried concerning future recurrence; therefore, I gave up on sexual function and chose total resection instead of nerve-sparing prostatectomy.”(P320)  F2.“Someone advised me to remarry a specific woman. However, this woman told me that she did not like a man who could not have an erection. Therefore, I gave up on remarriage.” (P3209)  F3.“I can tell my physician frankly that my sexual function is declining. It is encouraging because my physician cares about prescribing an erectile medicine.” (P3210) |
| Taro, et al(2022) | G1.Avoiding social interaction  G2.Lack of doctor-patient communication | G1.“Going out has become troublesome. For example, I have a lot of gatherings like alumni meetings, work activities. I have stopped going to those completely. I don’t even want to go outside anymore”(P423)  G2.“It can be difficult to discuss this with the physician, because of the atmosphere. I think that even if I have said anything, they wouldn’t be listening to me. They are always looking at their computer monitor.”(P423) |
| Koichiro, et al (2021) | H1.Increased family burden  H2.Inadequate awareness of bone metastasis  H3.Dissatisfaction with treatment outcomes | H1.“Exactly. All I want is to be able to live longer than my wife, even if it is only by one day, so that I can take care of her. That is my biggest wish. I have a fear of not being able to accomplish this. It is a top priority for me.” (P5113)  H2.“When I feel the slightest pain anywhere, I think, ‘is this another cancer?’ Or if my constipation continues, I think, ‘my colon is getting worse.’ I seem to be always worried about everything.” (P5114)  H3.“I was in shock. I didn’t expect for it to no longer be effective that soon. I believed that hormone therapy would be effective for a few years, so yes… I was shocked.”(P5113) |
| Wei, et al(2023) | I1.Efforts to resume normal work  I2.Insufficient doctor-patient communication  I3.Corporate support  I4.Support from coworkers | I1.“I did the [first] work for about eighteen months. I took a break for about six months and then I got the opportunity to [do the second work] ... I’d had a break from the hormone therapy at the time I started [the second work]…When I finished the project and I’d had to go back on the medication that I felt like I really sort of hit the wall and went into a bit of a slump.”(P5)  I2.“I don’t feel like I was adequately informed around the impacts of treatment, particularly the hormone therapy … I didn’t feel adequately prepared for the way the hormone therapy affected me … It affects your cognitive function so I felt like I couldn’t [work].”(P4)  I3.“There’s a limit to what you can do from home … working from home was probably ok three quarters of the time, but not all the time … It’s not just those things that department formally provided like the employee assistance program and a return-to-work plan and that sort of thing, that’s great, but as I said you can have a real impediment if your immediate boss isn’t supportive.”(P6）  I4.“We were able to work around the times when I had my immune system down, I was able to get support from other people within the group to carry out the work that I was going to do.”(P6) |
| Koichiro, et al (2020) | J1.Concerns about expected lifespan  J2.Insufficient understanding of the disease | J1.“I feel frustrated because there is no cure.”(P40)  J2.“I want to have more information about my disease and my options to enable me to discuss how to manage my condition with my doctor.”(P41) |
| Suzanne, et al(2018) | K1.Fear of the future, uncertainty  K2.Unwillingness to seek help  K3.Appeals being ignored  K4.Seeking solutions for side effects  K5.Shortage of medical resources in rural areas  K6. Economic burden of disease | K1.“So really, the next thing that faces me is death. Now, so, it was 4 months ago that my oncologist gave me, he said, ‘You’ll see your birthday.’ Well, that’s only 3 months away, it’s only 2 months away actually. What’s going to happen between now and then?”(P5)  K2.“Partly, I think, the reluctance of men to talk about their health, or to talk about personal issues that the idea of maleness, being able to be stoic and cope, type of thing. And just in the idea of maleness, that, sort of, men don’t cry, men are stoic, or you get on and cope with things, a lot of men have not been exposed to talking about personal matters and so find it difficult to do that.”(P5)  K3.“Openness, I think, for me for the treatment options. Probably the side effects were less discussed or less publicized. There was some, particularly in the radiation process, you could get burning, you could get itching, this, or that, but then later on you find out there was some others that they didn’t mention…On that score, I think there’s some minor explanation, you will get tired, you will get hot flushes, but no advice—what can you do about a hot flush; go and have a cold shower. There’s been nothing on that, and in terms of maybe a medication, is there something will help as it does for ladies, I’ve got no idea.”(P7)  K4.“And I think that’s—the big help that people need, and they are finding it here because they come to support group, it’s looking for some comfort, I think, or some advice, or to talk to someone who’s been there and done that. I think that a lot of trouble with the GPs and the surgeons, they—a lot of them don’t have that first-hand experience, even though they work with it every day of their lives they don’t—they haven’t had the actual experience of living with the disease first-hand anyway.”(P8)  K5.“Well I’m up in the country and when I got diagnosed there was really no services in my town and it was a, you know an hour and a half trip to the closest place where could get anything done…Well they certainly—they didn’t even have chemotherapy services here. And they didn’t have helpers such as prostate nurse etcetera, people that were specifically trained in this sort of cancer. My oncologist, my urologists are all down in Melbourne. I mean they’re 2 hours away in a motor car.”(P7)  K6.“It’s about $40 000 a year. I think it’s about 4 to 12, about $4000 a month I think. Which the majority of people can’t afford anyway. So he’s got a number of other drugs, too. But that’s the one he’d like to use next for me. So we’ve got to deal with that when we get there.”(P7) |
| Claire, et al(2020) | L1. Anxiety and fear  L2. Low Mood and depression  L3. Accept life-prolonging treatment | L1.“I was worried, metastasis means that it’s spreading.” (P3096)  L2.“Yes [patients symptoms affect me], they lower my spirits.” (P3097)  L3. “I’ll take everything that helps me. That’s my attitude.”(P3099) |
| Stefan, et al(2019) | M1.Changes in daily activities  M2.Keep a positive attitude  M3.Urinary frequency affects sleep quality | M1.“I’m not the same man physically that I was a year, a year and a half ago. There’s things like playing golf or playing basketball, walking long distances, that I just can’t do now.” (P62)  M2. “That was a huge shock for both of us to realize what the mortality situation was and emotionally that was very hard to deal with for me and for her. But as time has developed over the past year especially and the increasing bad news as far as my diagnosis and prognosis has been going on, I’ve kind of gone through another emotional change as far as living life to the extremes and to the fullest, so I’m not willing to settle for just being an old guy and crawling up in the bed in the fetal position.” (P62)  M3. “That’s also something that interrupts my sleep at night, maybe once or twice a night I get up. On a good night, I just get up once. Most of the time I have to get up, like, twice. Even though I’ve tried stuff like making sure I don’t drink anything past say 8:30 at night, and I still have to get up.” (P61) |
| Erin, et al (2017) | N1.Feelings of depression and anxiety | N1.“Treatment-associated emotional turmoil(anxiety/frustration).”(P574) |
| Shucheng , et al(2022) | O1:Limited decision participation  O2: Need disease knowledge and option autonomy | O1.“I just followed medical advice. … I am unaware of the specifics of the treatment. These doctors are experienced and professional.” (P4193)  O2.“I genuinely want to be involved in my disease treatment, but no one informed me, and I’m still puzzled. What alternatives do I have for treatment? Which is the more radical and which is the more conservative? … So that I am aware of what to expect. … In any case, whatever kind of therapy is more suited for me now and in the future? I still want more information.” (P4193) |
| Tong, et al(2022) | P1.Adaptation of sexual behaviors and intimate relationships  P2.Inadequate sexual health support | P1(1).“We don’t need to talk about this, as she knows my situation [erectile dysfunction]…. Our relationship is good so that we don’t need any communication. Anyway, she understands this.”(P3088)  P1(2).“I think we became more intimate after the disease. Before I had cancer, we were used to not communicating very often. Now, I am glad that she takes care of me a lot. I even think about how I can repay her. I also express my concerns now and sometimes try to do some housework to relieve her.”(P3089)  P2.“I see the doctor once a month. Sometimes when I see him, I would like to ask if there is any way to solve my sexual problems. But I feel too embarrassed to ask him.”(P3089) |
| Ulrika, et al (2022) | Q1.Uncertainty about disease progression  Q2.Worrying about my family's future | Q1.“I don't know what it can be, if it's my hip joint, or if it's …, one feels uncertain if it's the cancer that has spread.”(P4)  Q2.“… then I become sad [sobs] … that we have to move away from here … if I don't make it how will she manage.”(P6) |
| Sandra, et al(2020) | R1.Consider life-prolonging treatment  R2.Reflection on life's end  R3.Fear of uncontrolled symptoms at the end of life | R1.“In no way have I imagined that I will all of a sudden be completely cured. But … that … well, that I can … keep going and feel well a little longer than I would otherwise have done.” (P3)  R2.“No, I can't say I do. It's on and off, off and on. But no, not a lot. In some weird way I keep it at a distance. But sure it comes to mind sometimes …”(P4)  R3.“So of course you think about … what the end will be like, if it'll be the pump [heart] that stops or if it's … a prostate cancer death, it's not that merciful, you've read many gloomy stories about that.”(P4) |
| Abbas, et al (2023) | S1. Fear of not being fully cured  S2. Fear of cancer recurrence  S3. Efforts to adjust lifestyle to cope with the disease  S4. Relying on spiritual faith | S1.“When the doctor saw the pathology result, he said that there are still cancer cells. When I heard this, I felt that my death was near and that my cancer would not be cured.”(P6724)  S2.“When I went to perform this test [PSA] and even close to the time of the test, I was very worried about what the result of this test would be… this worry between taking this test and awaiting to get the result and show it to the doctor became much more and I had stress.”(P6725)  S3.“I read a lot on the internet about the things that should be done to prevent cancer recurrence, and I tried to incorporate them into my life if they made sense.”(P6727)  S4.“I left everything to God and I think this is the best thing that will reduce my worries about cancer recurrence.”(P6727) |
| David, et al(2022) | T1. Redefining masculinity  T2. Building a sense of control  T3. Maintaining social connections | T1. “I do feel like less of a man but have learned to be comfortable in my own skin. I’ve become less embarrassed about having had cancer, it may come back…I’m going to have to move on after that. Exercise can, it can help.”(P427)  T2.“Off the top of my head, probably exercise mainly for taking back or holding onto what the cancer took from me. You have to work harder to hold onto what remains. That makes me feel empowered as a man.”(P425)  T3.“So we advocate for men and prostate cancer and, of course, we have monthly meetings every second Tuesday so I started to go to those, and I knew one of the participants there and so I ended up getting on the board of directors.” (P426) |
| I B Van, et al(2018) | U1.Afraid of chemotherapy  U2.Worried about PSA test results  U3.Rreluctant to tell children  U4.Supported by grandchildren  U5.Dissatisfied with nursing staff | U1.“Then they offered me chemo. But I always said I won't do chemo, I'd rather be dead. But, uh, if you have to face it, you see it differently. I said yes… I woke up in the morning and wanted to comb my hair. I had lost much hair. … And I was sick, you just don't want to know. I lay in bed terminally ill. … The third time I told the professor that I quit. I am much too ill, and I said, you know what it is … we all know I am going to die. what are we doing? Yes, lessen the pain, but I hardly have any pain at all.”(P58)  U2. “And well, yes, then I had to go back to doctor X, and he showed me on screen everything that was going on. Well, that was not funny. … All of my bones were affected.”(P9)  U3.“I went to the hospital for a day or three, then I came home. I told my wife, “We are not telling the children anything because they may start worrying.” So instead, I told her, “It's no problem, I feel fine, 100 percent. DO NOT tell the children.”(P59)  U4. “Ah, that makes you emotional eh, yes, that such a child eh, that it engages him. … Yes, that I think, I, well, …then we were … anyway he starts walking next to me, and he says, grandpa, do you still have cancer?”(P60)  U5.“…and I thought that a nurse practitioner was someone who, well, who sympathized with you a bit, provided care, checked whether quicker treatments were possible. Well, the name says it all, practitioner. But she was never there.”(P60) |
| Siv, et al(2021) | V1:Need for information and support  V2:Seeking peer support | V1.“The urologist said I could call if I had any questions after the consultation. I called, several times, but never got in touch. I was very disappointed. It felt like an assembly line because a doctor is so busy and unavailable.”(P1658)  V2.“The most important thing throughout the process has been talking to others in the same situation, how others have experienced getting prostate cancer and side effects…several I have met who have had prostate cancer ask me ‘how are you doing?’, I think it's very reassuring to talk to others who have been through the same.”(P1659) |
